# Changes in Frequency and Mode Shapes Due to Damage in Steel–Concrete Composite Beam

**DOI:** 10.3390/ma14216232

**Published:** 2021-10-20

**Authors:** Małgorzata Jarosińska, Stefan Berczyński

**Affiliations:** 1 Faculty of Civil and Environmental Engineering, West Pomeranian University of Technology in Szczecin, al. Piastów 50a, 70-311 Szczecin, Poland; 2 Faculty of Mechanical Engineering and Mechatronics, West Pomeranian University of Technology in Szczecin, al. Piastów 19, 70-310 Szczecin, Poland; Stefan.Berczynski@zut.edu.pl

**Keywords:** damage diagnosis, structural health monitoring, non-destructive testing, modal parameters, steel–concrete composite beams

## Abstract

This study presents an analysis of changes in the vibration frequency and mode of vibration of a composite beam due to damage. A steel–concrete composite beam was considered, for which numerical analysis (RFE model) and experimental tests were conducted. Two levels of damage were introduced to the beam. To determine the changes in the mode of vibration before and after the damage, the modal assurance criterion (MAC) and its partial variation (PMAC) were applied. The curvature damage factor (CDF) was used to determine the changes in the modal curvature. The natural frequencies were sensitive to the introduced damage. The results show that the MAC is not effective in determining the location of damage in the connection plane. Two different coefficients were introduced to locate the damage. The PMAC was used for sections of subsequent modes of vibration and allowed effectively locating the damage. The CDF considered simultaneous changes in the curvatures of all vibration modes and was effective in locating the damage in the connection plane.

## 1. Introduction

Steel–concrete composite beams are structural members that typically consist of a steel I-beam and a concrete slab joined together by connectors which ensure cooperation between both parts. The beams are often used as the main girders in bridge construction. The main task of the connectors in composite members is to create a uniform cross-section between the steel and concrete. Thanks to the tensile strength of the steel and the compressive strength of the concrete, it is possible to build structures with extended spans.

The transport sector has seen rapid increase in recent years. The trucks used for moving goods are often overloaded and driven with limit-exceeding speed. This causes an intensive exploitation of existing roads and bridges. Periodic inspections are not always sufficient for bridges, which are considered as complex and strategic structures. The inspections primarily consist of visual assessment of the object. As mentioned in [1], the technical condition of the structure during the inspection is determined based on an expert’s opinion, which is subjective and therefore susceptible to a certain level of uncertainty. It should also be noted that there may be difficulties in determining the internal damage. In the case of composite structures, damage in the connection zone may not be detected during the inspection. Partial delamination of the steel and concrete connection zone may result in corrosion of the connectors. Deterioration of the connectors over the years reduces the cooperation between the steel and concrete. This affects the load-bearing capacity of the structure and is crucial for the safe use of the building. Therefore, continuous structural health monitoring (SHM) is becoming more and more common [2,3,4,5,6,7]. Virtually all large and important bridges are now equipped with monitoring systems [8]. Continuous monitoring systems are based on so-called non-destructive testing (NDT) [9,10,11,12]. Such systems increasingly use modal analysis. It consists in registration of vibrations of the structure, caused by time-varying loads, and analysing its modal properties. Occurring damage causes disruption to the proper operation of the structure. It is reflected in changes in modal properties, i.e., natural frequencies, modal damping ratios and mode shapes. Methods based on the analysis of those changes can be an effective tool in health monitoring. Finding a fully effective method is difficult because the sensitivity of modal parameters is influenced by many factors, not only those related to the damage itself. Environmental conditions, e.g., temperature, wind, humidity or boundary conditions and loads, can also have an effect [13,14,15]. For several decades, researchers around the world have been searching for an effective method of damage diagnosis. Studies were conducted for various types of structures, materials or damage. There are a number of older [16,17] and newer [18,19,20] studies that analyze this issue. A detailed review of studies on damage diagnostics conducted between 2010 and 2019 was performed by Hou and Xia [21]. Their paper presented methods for vibration-based damage identification of structures, divided them into different damage indices and analytical/numerical techniques used and listed their advantages and disadvantages. The authors of the paper cited 292 publications, which shows how many academic centers around the world conduct research on this issue. However, no universal method which would allow identifying all types of damage for various types of structures has been developed. Therefore, there is a need to develop a method which would be applicable not only in computer calculations or laboratory conditions but also primarily in real-life structures based on vibration measurements.

Natural frequency is the most commonly analyzed modal parameter in the aspect of damage detection. With regard to steel–concrete composite beams, analysis of the changes in natural frequencies due to damage in the connection plane was carried out by researchers from Italy—Dilena, Morassi and Rocchetto. A two-part study [22,23] from 2003 presented the results of experimental [23] and analytical [22] tests performed for steel–concrete composite beams with different degrees of connection. Damage which was applied to the connection plane by removing the concrete around the studs on one or both ends of the beam was analyzed. The researchers found that axial vibration frequencies are not affected by the damage, unlike the flexural vibration frequencies, which show significant susceptibility. In 2004, Dilena and Morassi [24] presented the results of another experimental study performed on steel–concrete composite beams. The geometry of the beams and stud location were based on a previous study [23]. The study presented different types of damage to the connection plane. The concrete around the stud was removed at one end of the beam, the stud was cut to a certain length and the space was filled with concrete. The results showed that the axial vibration frequency is not sensitive to damage in the connection plane of the steel–concrete member. The flexural vibration frequencies showed a higher sensitivity, especially for beams with a partial connection. Significant differences were observed for higher frequencies and more extensive damage. Experimental results were verified using an analytical model [25].

Often, an analysis of changes in mode shapes is performed in conjunction with the analysis of natural frequency changes. The damage found in the structure causes a local change in the stiffness of the system, which is associated with a local change in its vibration mode. The modal assurance criterion (MAC) is used to compare the mode shapes of vibration before and after the introduction of damage [26]:

(1) $$\mathrm{MAC}=\frac{{\left|Q_{A}^{T}Q_{B}\right|}^{2}}{\left(Q_{A}^{T}Q_{A}\right)\left(Q_{B}^{T}Q_{B}\right)}$$

where $Q_{A}$ and $Q_{B}$ are the mode shape vectors that are being compared. The MAC determines the degree of correlation between a pair of vectors taking values 0 ÷ 1. When both vectors are perfectly matched, the MAC equals 1. Values below 1 indicate a mismatch between two vectors, which may be a sign of damage. The same conclusions were presented in the study of [27], which showed the results of tests of a steel–concrete composite girder. It was shown that the method can be used to evaluate the local fatigue failure of the composite girder. Many variations of the MAC can be found in the literature [28]. One of them, which can be used to determine the location of damage, is the partial modal assurance criterion (PMAC) [29]. It is calculated similarly to the MAC, where a pair of mode shape vectors before and after damage is compared, but is limited, for example, only to the analysis of a part of the mode. The method is, however, rather unpopular. The most common variation of the MAC is the COMAC (coordinate modal assurance criterion) [30], which can also be used for locating the damage. The COMAC determines the correlation between mode shapes for one selected node and is determined as a sum of several modes of vibration. However, it turns out that MAC/COMAC ratios may not be sensitive enough to locate the damage or show false results [31]. Therefore, a method based on the change in curvature (second derivative) of the mode shape for the pre- and post-damage conditions is often used for locating the damage. This method may prove to be more effective due to the direct relationship between the curvature and stiffness, which changes locally due to damage. For real structures, the curvature of the mode shape is determined directly from its shape according to the relation [32]

(2) $$MC_{p}=\frac{\left(y_{p+1}-2y_{p}+y_{p-1}\right)}{h^{2}}$$

where $y_{p}$ is the vibration mode’s component at point p, and h is the distance between two adjacent measurement points. The first demonstration of the effectiveness of this method was presented by Pandey et al. [32] on the basis of numerical tests performed on two beams—cantilever and free-supported. The damage (stiffness reduction) location coincided with the area of maximum curvature changes. Additionally, the changes in the curvature were more extensive with increasing damage. The MAC and COMAC coefficients were also analyzed but were not significantly susceptible to the introduced damage. Analyses for a steel and concrete composite structure were presented by Liu and De Roeck [33]. Numerical analyses performed on a high-speed railroad bridge in Italy showed a significant sensitivity of the curvature of the vibration mode to damage in the connection zone for a MAC value of 1.0.

However, the method of damage detection based on curvature analysis of the vibration mode is less reliable when applied to structures with extensive damage. It is therefore necessary to analyze each vibration mode to be able to effectively localize every damage location. In such cases, the CDF parameter is more reliable. It simultaneously takes into account the curvature changes for several vibration modes and is defined by the relation [34]

(3) $$CDF_{p}=\frac{1}{N}\overset{N}{\underset{n=1}{\sum }}\left|MC_{p,n}-MC_{p,u}\right|$$

where N is the number of vibration modes, and $MC_{p,n/p,u}$ is the curvature of the mode shape in point *p* for the damaged/undamaged state. Chandrashekhar and Ganguli [35] presented the validity of using the CDF as an indicator of both single and multiple damage for a cantilevered beam. Datta and Talukdar [36] carried out numerical simulations for a bridge model based on which they concluded that for multiple damage, the CDF allows unambiguously locating the damage. The review of studies has shown that the effectiveness of the modal curvature method depends on the density of the measurement mesh [34,37]. Therefore, when using this method, it is important to select a sufficiently dense mesh of measurement points to accurately determine all damaged areas.

In summary, different damage identification methods have been developed over recent decades. However, a method that is effective and yet applicable to real-life structures is still to be developed. With the strong development of steel–concrete composite structures, there is an urgent need to develop an effective damage identification method for this type of structure using measured data. A method for a fast, non-destructive evaluation should be developed for practical purposes.

The aim of this study was to assess the applicability of selected non-destructive damage detection methods to diagnose damage of steel–concrete composite beams. Beams of this type are very often used as the main load-bearing girders of bridge structures. The internal damage that may occur in such composite beams is invisible, carrying a safety hazard during the use of the structure. This paper analyzed damage to the connection zone in composite beams. The analysis of the sensitivity of modal parameters to damage in the composite beam began with the analysis of changes in parameters determined globally—for the entire beam. The changes in natural frequencies and mode shapes were analyzed using the MAC. The following step was to locally determine parameters for sections of the beam. The vibration mode vector was analyzed using the PMAC, while the modal curvature was determined using the CDF. As the damage was introduced locally, the stiffness of the beams also changed locally. This is visible in the changes in some parameters, allowing for pin-pointing the location of the damage.

## 2. Experimental Research

**Figure 1:**
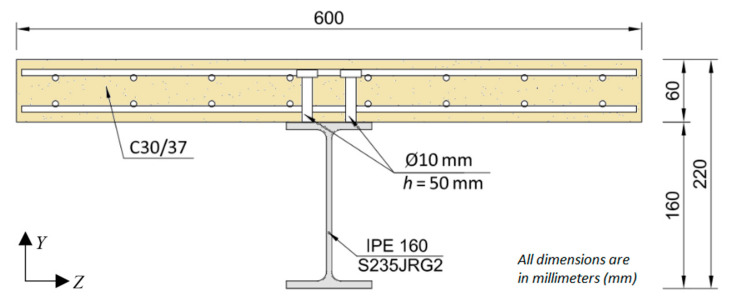
Cross-section of the studied beam.

**Figure 2:**
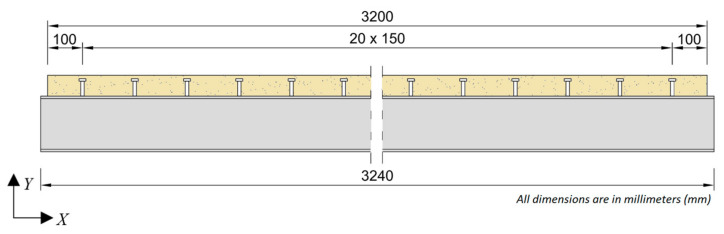
Longitudinal section of the studied beam.

The studied beam was a steel–concrete composite with an IPE 160 steel I-beam made of S235JRG2 steel and a 600 mm-wide, 60 mm-thick slab made of C30/37 concrete. The length of the beam was 3200 mm; however, the steel I-beam protruded by 20 mm on each end, for technical purposes. The beam was assembled using steel-headed studs with a total height of 50 mm and a diameter of 10 mm. The studs were spaced in pairs every 150 mm along the length of the beam, with the first pair located 100 mm from the edge of the beam. The cross-section and longitudinal section of the beam are shown in Figure 1 and Figure 2, respectively.

**Figure 3:**
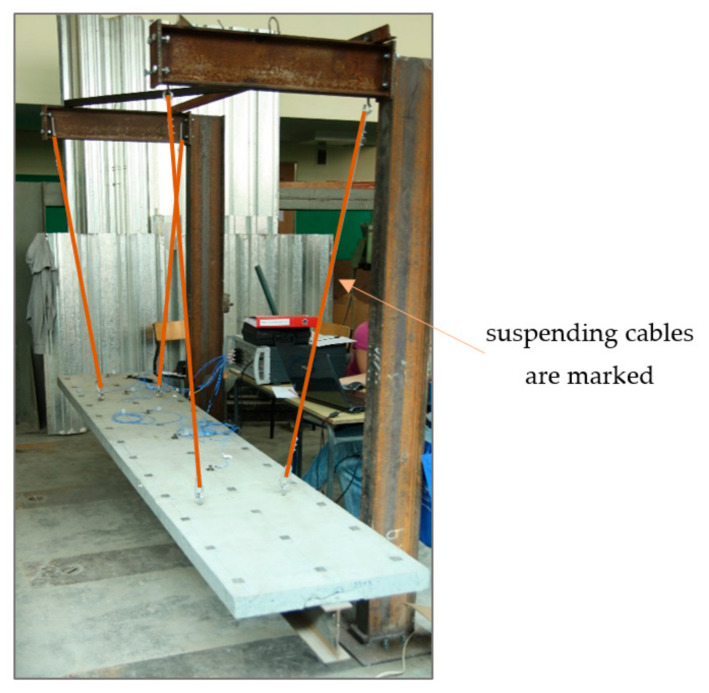
Experimental test stand.

Experimental tests were carried out on the test bench presented in Figure 3. This setup allows neglecting the effect of support susceptibility on the results, making it easier to analyze them and identify the missing parameters for the computational model. In order to achieve this, the beam was suspended from a steel frame by four flexible steel cables. The fixing points on the beam were determined so that they coincided with the theorical nodes of the basic (first flexural) mode of freely supported beam vibrations. A similar setup can be found in other studies [24,38,39,40].

**Figure 4:**
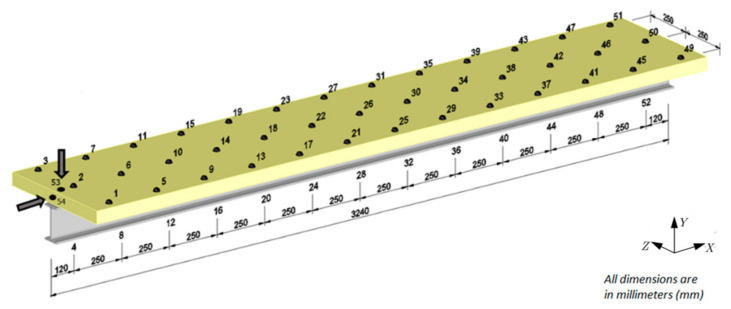
Measurement and excitation points.

The experimental tests were performed for 52 measuring points along the surface of the composite beam, as shown in Figure 4. Two additional points, 53 and 54, were located at one end of the beam. These points were used to excite the beam using a modal hammer. At point 53, the force was applied perpendicular to the concrete slab (along the *Y*-axis) to induce flexural vibrations of the beam. To induce axial vibrations of the beam, the excitation force was applied from the front of the beam at point 54—along the *X*-axis.

**Figure 5:**
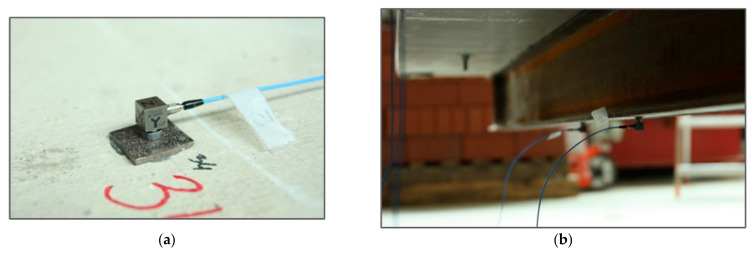
Acceleration sensors: (**a**) top surface of the slab; (**b**) bottom surface of the I-beam.

Accelerations in three orthogonal directions were recorded using piezoelectric acceleration sensors (Figure 5). A multi-channel LMS SCADAS III analyzer was connected to a computer with LMS Test.Lab 13A (2015) software from LMS International and was used to measure the signals. The Polymax algorithm available in Test.Lab software was used to determine the model’s modal parameters.

In order to find the flexural mode of vibrations, which will be analyzed later in this study, only points located in the median plane (2, 4, 6, 8, …, 52) were used. This allowed analyzing the beam as a 2D model.

**Figure 6:**
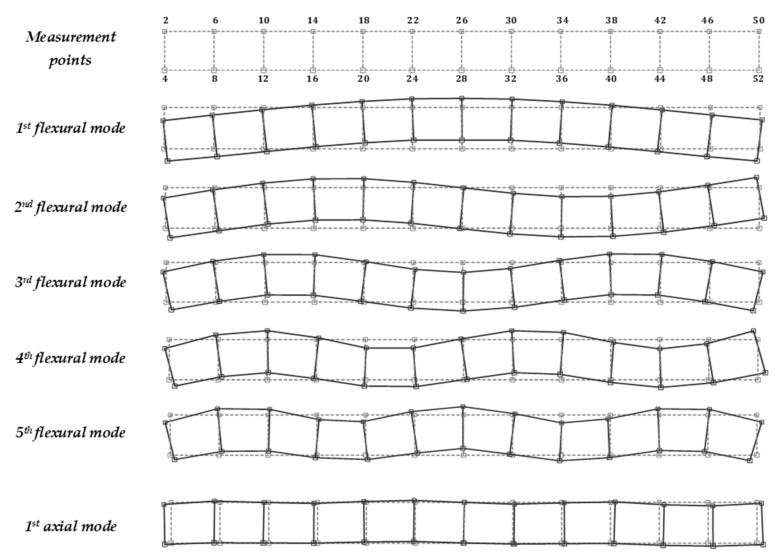
Mode shapes of vibration obtained in the experimental tests.

Table 1 shows the natural frequencies determined during the experimental tests (five flexural and one axial) and the corresponding mode shapes determined for the beam median plane—Figure 6.

**Figure 7:**
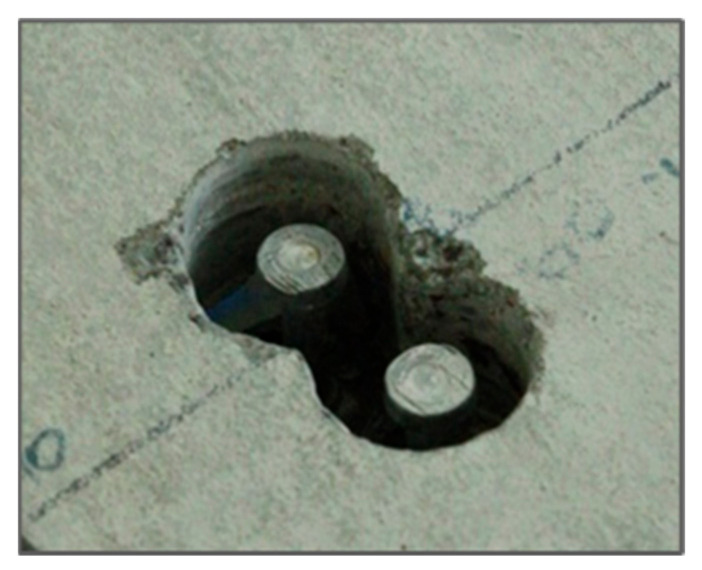
Damage to the connection.

The next step of the experimental tests was to introduce damage. Damage to the connection was introduced by removing the concrete around a pair of studs to eliminate the cooperation between the steel and concrete (Figure 7).

**Figure 8:**
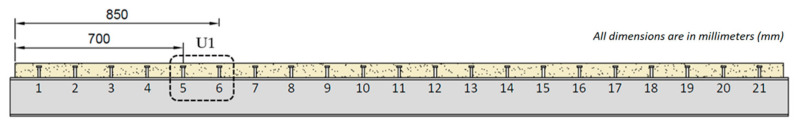
Damage U1.

**Figure 9:**
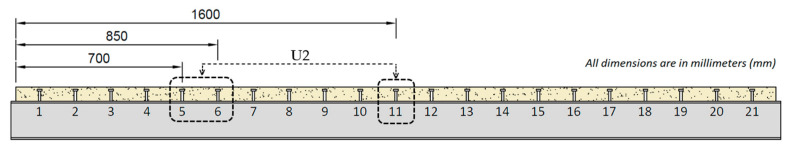
Damage U2.

Two levels of damage were introduced, labeled as U1 and U2. In the first level (U1), concrete was removed around two adjacent pairs of studs—numbered 5 and 6 (Figure 8). The U1 damage was then extended by removing concrete around stud pair number 11 located in the middle of the beam. This stage of damage was labeled as U2 (Figure 9). The frequency and natural vibration mode of the beam with the applied damage were measured in the same way as for the beam without damage (which was described earlier).

## 3. Beam Model

**Figure 10:**
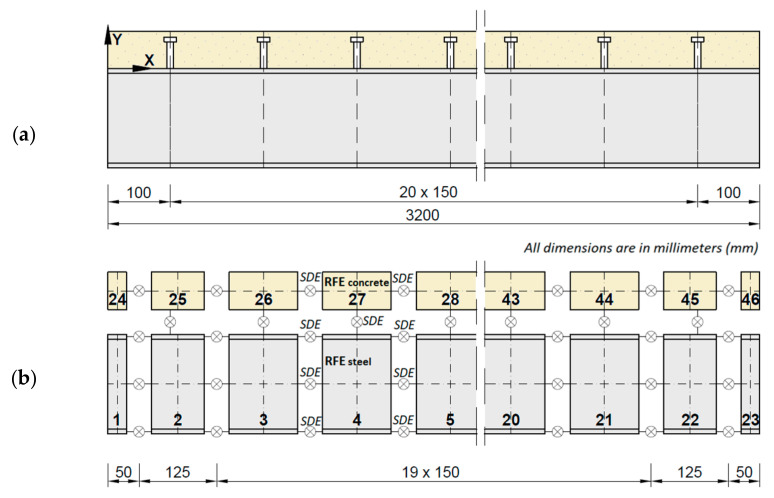
RFE model of the beam: (**a**) primary division; (**b**) secondary division.

A numerical model of the studied beam was prepared. A proper model allows verifying the obtained results of the measurements and analyzing the simulation of the influence of various parameters on those results. The analyzed steel–concrete composite beam was modeled using rigid finite elements (RFEs) as a 2D model. Taking into account the discrete arrangement of the stud pairs on the beam, in the first step (so-called primary division), the beam was divided into segments (Figure 10a). In the second stage (so-called secondary division—Figure 10b), a spring-damping element (SDE) was placed in the center of each section to concentrate its spring and damping properties. Successively adjacent SDEs were connected by non-deformable solids called rigid finite elements (RFEs) [41,42].

The steel and concrete parts of the beam were considered separately when creating the beam model. The connection between adjacent RFEs used for modeling of the concrete slab was performed in a standard way—with a single SDE placed in the axis of the slab. For the steel part, a different approach was proposed—a single SDE was replaced by three SDEs placed in the axes of the web and flanges. In this way, the spring-damping properties of the whole section were distributed between the two flanges and the web. In summary, the beam model consisted of 46 RFEs—23 for the steel I-beam and 23 for the concrete slab. Figure 10 presents the beam model with all of the RFEs and SDEs.

To determine the model, it is necessary to know the parameters that characterize the steel I-beam, the concrete slab and the connection. Some of the parameters, e.g., longitudinal/transverse modulus of elasticity of steel, density of the materials, Poisson’s ratios or cross-sectional areas of elements, were determined on the basis of measurements taken during experimental tests and data from the literature. The characteristics are presented in Table 2.

During the development of the model, it was also necessary to determine the parameters describing the stiffness of the composite beam, i.e., the longitudinal modulus of elasticity of the reinforced concrete slab *E_c_* and the stiffness of the connection in both directions *K_X_* and *K_Y_*. These parameters are determined in parametric identification. It should be mentioned that *K_X_* and *K_Y_* are the stiffness of the stud pair. The determination required a complete match of the first frequency of axial vibrations determined numerically and experimentally. The longitudinal modulus of elasticity of the concrete slab *E_c_* is highly correlated with this mode of vibration. At the same time, the values of the first five frequencies of flexural vibrations determined numerically and experimentally were compared and matched. The identification process was conducted with a MATLAB optimization toolbox. The results of the stiffness parameter identification are presented in Table 3.

A comparison of the natural frequencies obtained from the experimental tests and the calculated natural frequencies is presented in Table 4.

As shown, a high compliance of the results of flexural vibration frequencies was achieved. The maximum difference between the values obtained from experimental tests and from the model was approximately 1%. Complete matching of the axial vibration frequencies was required by the initial assumptions. The axial vibration frequency was used only to identify the beam model and will not be analyzed further in this paper.

The compliance between the mode shapes obtained from the model and experimental tests was determined on the basis of the MAC in accordance with (1). The MAC takes the scalar values of 0 ÷ 1, where the higher the result, the better the correlation. Table 5 shows the MAC values for vectors of the mode shapes obtained from the experiment **Q_A_** and the model **Q_B_**. The model can be assumed as reliable when the MAC value is higher than 0.8 [43,44]. As it can be seen, for the first four modes of vibrations, MAC > 0.8 was obtained. Thus, it can be concluded that there is a good compliance between the model and the experimental results. As the MAC for the fifth vibration mode was 0.72 < 0.8, it was decided not to include it in the next stage of the study.

**Figure 11:**
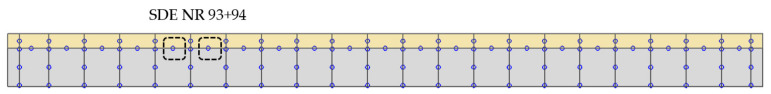
Modified SDE for damage level U1.

**Figure 12:**
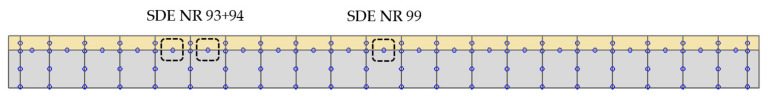
Modified SDE for damage level U2.

The next stage of the numerical analyses was the simulation of damage to the connection. The simulation of damage to the connection was performed by changing the spring properties of the SDE used for modeling the connection. Null stiffness coefficients were assumed for certain SDEs—*K_X_* = 0 and *K_Y_* = 0. The locations where damage was simulated corresponded to those of the experimental tests. Figure 11 and Figure 12 present the beam models where the modified SDEs for damage levels U1 and U2 are shown.

## 4. Results

### 4.1. Natural Frequencies

Changes in natural frequencies as a result of the introduced damage to the connection, determined both experimentally and numerically, are presented in Table 6 and Table 7.

As shown, a high compliance of results was obtained for frequency changes due to damage determined numerically and experimentally. The changes in frequency due to damage to the connection reach between 1% and 6%. Higher values were obtained in the experimental tests. The most sensitive to the damage was the third mode of vibration. Maximum changes for this mode were (1) U1 − Δ*f*_3_*_, exp_* = −4.8% and U2 − Δ*f*_3_*_, exp_* = −5.83% for the experimental results, and (2) U1 − Δ*f*_3_*_, num_* = −3.57% and U2 − Δ*f*_3_*_, num_* = −4.56% for the numerical model. For each individual measure, there was a decrease in the vibration frequency as a result of the damage, which is related to the loss in the beam’s stiffness.

### 4.2. The Modal Assurance Criterion (MAC)

The MAC has many applications and is commonly used in modal analysis. At the beginning of the analysis, it was used to determine the degree of conformity between the numerical model and the real-life beam. Here, the MAC was used to compare the vibration mode shapes before and after introducing the damage. The MAC was determined in accordance with (1), with the vectors **Q**_A_ and **Q**_B_ being the vibration mode before and after the introduction of damage, respectively. The MAC was determined only for the vertical components of displacements—along the *Y*-axis. Due to the nature of the beam operation, flexural vibrations are the most significant. The displacements of points in the direction perpendicular to the beam axis (*Y*-axis) are much larger than those along the other axis (*X*-axis). Therefore, in the analyses, it was decided to omit the horizontal displacements.

To analyze the results, the MAC was presented as a percentage value, as shown below:

(4) $$\Delta MAC_{i}=\left(1-MAC_{i}\right)\cdot 100\%$$

Table 8 presents the changes in the shape of the vibration mode using the MAC for numerical and experimental data.

As it can be seen, the maximum MAC change was 5.49% for the experimental results, fourth mode of vibration and U2 damage. For the numerical results, the same change was only 2.66%, which is a significant difference. It should also be noted that the MAC changes observed for the first two modes of vibration (both for numerical analysis and the experiment) did not exceed 1%. Therefore, it can be concluded that these modes are not sensitive to changes in the beam’s connection. It should also be emphasized that the increase in the damage did not increase the MAC changes—in most cases, they were smaller. These analyses lead us to conclude that the MAC did not prove to be an effective identifier of connection plane damage in the analyzed composite beam.

### 4.3. The Partial Modal Assurance Criterion (PMAC)

The analysis of local shape changes of the vibration mode was carried out based on the PMAC, which was determined analogically to the MAC in accordance with (1). The difference was that the PMAC was determined for fragments of vectors of consecutive vibration modes. This parameter, similar to the MAC, was determined for the displacement in the Y direction with the omission of the X direction. It should be emphasized that the division into segments was carried out individually for each mode shape. This allowed covering the area of large curvature while avoiding the mode nodes. The analyses were started from the second mode of vibrations.

Because the steel and concrete parts of the beam were considered separately when modeling, mode shape vectors of the RFEs were also obtained separately. Therefore, a vector consisting of 23 points for the concrete part and 23 points for the steel part was obtained. The mesh of measurement points, for which the mode shapes were determined, consisted of 26 points—13 for the concrete part and 23 for the steel part.

**Figure 13:**
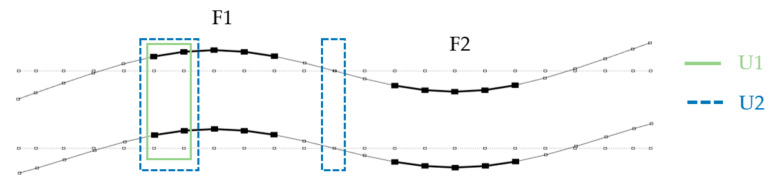
Second mode shape with sections analyzed with the PMAC and location of damage.

**Figure 14:**
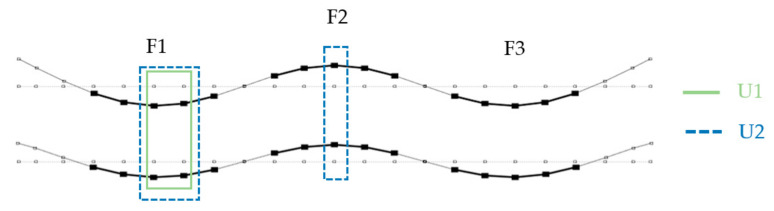
Third mode shape with sections analyzed with the PMAC and location of damage.

**Figure 15:**
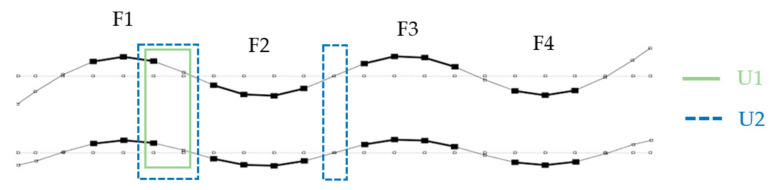
Fourth mode shape with sections analyzed with the PMAC and location of damage.

Figure 13, Figure 14 and Figure 15 show the analyzed mode shapes for the undamaged beam with selected sections used in PMAC analysis. The figures also present the location of the damage to the connection.

Because the mesh of measurement points for the conducted experimental tests did not conform with the RFE nodes in the model, it was necessary to perform a transformation of the experimentally determined mode shape from the sensor locations to the RFEs. Interpolation of the experimental vibration modes was performed. As a result, the experimentally determined mode shape vector contained 23 points from the concrete part and 23 points from the steel part. The fragments for which the PMAC was determined were consistent with the RFE nodes in the model.

The local changes in the mode shape based on the PMAC are expressed as

(5) $$\Delta PMAC_{i}=\left(1-PMAC_{i}\right)\times 100\%$$

**Figure 16:**
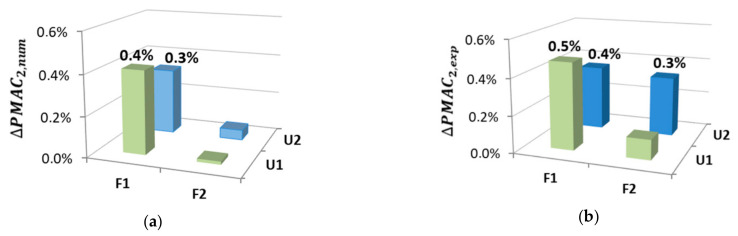
Local mode shape changes based on the PMAC for the 2nd vibration mode: (**a**) numerical results; (**b**) experimental results.

**Figure 17:**
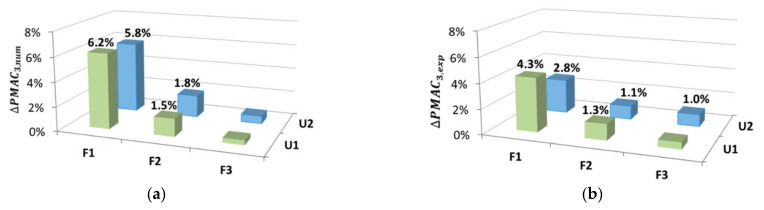
Local mode shape changes based on the PMAC for the 3rd vibration mode: (**a**) numerical results; (**b**) experimental results.

**Figure 18:**
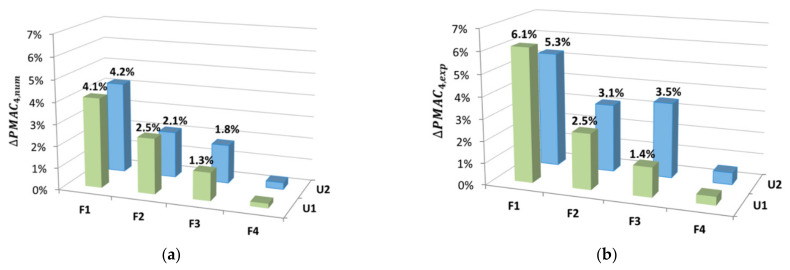
Local mode shape changes based on the PMAC for the 4th vibration mode: (**a**) numerical results; (**b**) experimental results.

Figure 16, Figure 17 and Figure 18 show the local changes in the mode shape due to damage to the connection, determined in accordance with (5) for both numerical and experimental data.

The results of the conducted experimental tests and numerical analyses indicate that this method allows effectively locating the damage in the connection plane of the beam. The damage location was clearly visible thanks to the third and fourth mode shapes. The second mode of vibration turned out to be the least sensitive to changes. The method is relatively simple for analysing as it is based only on the analysis of changes in vibration form vectors, which is definitely its great advantage.

### 4.4. Mode Shape Curvature

**Figure 19:**
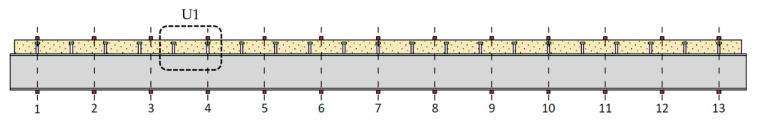
Location of sensors along the beam, with U1 damage shown.

**Figure 20:**
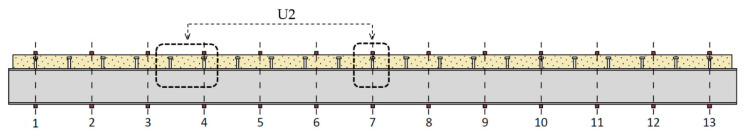
Location of sensors along the beam, with U2 damage shown.

The curvature for successive points of the mode shapes for the steel and concrete was determined in accordance with (2), taking into account only the *Y*-axis displacements. The analyses also excluded the extreme points at the edges of the beam. The curvature for the experimentally determined mode shapes was determined for the actual measurement points, without using prior interpolation as in the PMAC analysis. Each interpolation was subject to certain errors, which, in the analysis of the shape of the mode of vibration, may have a small influence, but in the case of the curvature, the differences can be significant. Therefore, in this case, the points of the vibration mode are the same as the points of the measurement mesh. As the number of measurement points along the beam was 13 + 13, a mode vector consisting of 13 points for the concrete slab and 13 points for the steel I-beam was obtained. Figure 19 and Figure 20 show the location of the sensors along the beam axis with the U1 and U2 damages.

**Figure 21:**
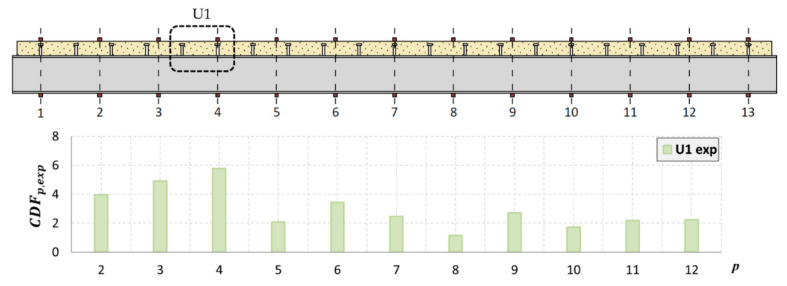
The CDF for damage level U1 in a real-life beam.

**Figure 22:**
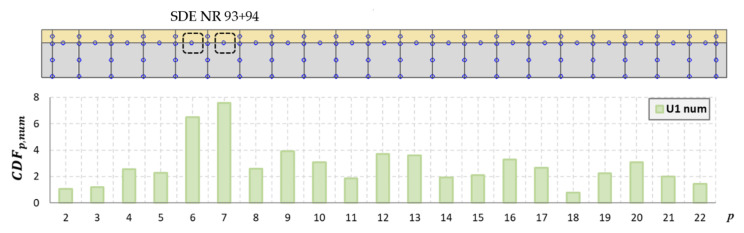
The CDF for damage level U1 in a model beam.

**Figure 23:**
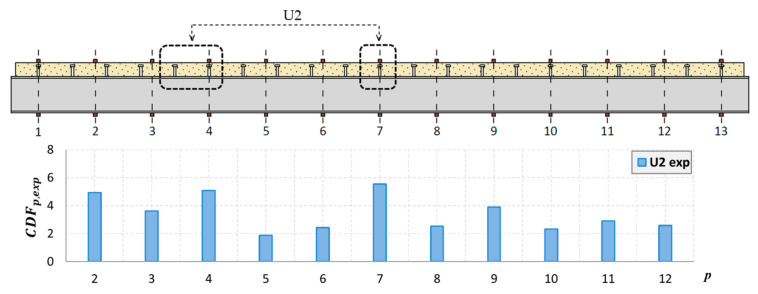
The CDF for damage level U2 in a real-life beam.

**Figure 24:**
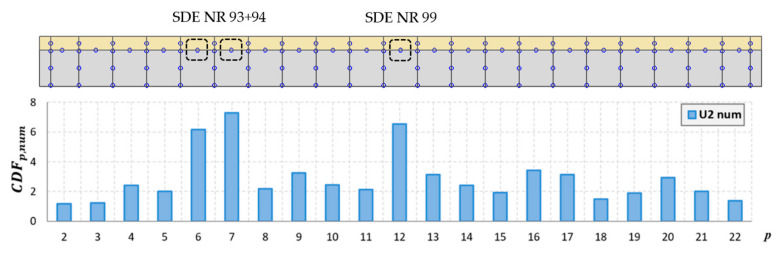
The CDF for damage level U2 in a model beam.

Because it is not possible to indicate the place of damage for every mode shape based on the analysis of its curvature, the CDF determined in this study takes into account the curvature of multiple modes simultaneously. This coefficient was determined for the analyzed mode shapes for both the steel and concrete. The method based on the CDF is relatively easy to interpret because only a single curve is analyzed, which significantly reduces the time of analysis. The results of the numerical and experimental modes are presented for damage levels U1 (Figure 21 and Figure 22) and U2 (Figure 23 and Figure 24).

As shown, based on the CDF analysis, it is possible to effectively indicate the location of the damage in the studied beam. In the case of numerical analyses, the CDF plot precisely indicates the location of damage, both for the U1 and U2 damage levels. For the experimental results, the values of the CDF were the highest in the location of the damage; however, the results were not that unambiguous. It can be assumed that this is related to the use of a different measurement mesh density for the experimental and numerical tests. Therefore, it can be assumed that with the use of a larger number of sensors during the experimental tests, the indication of the damage location would be more distinct.

## 5. Conclusions

This study analyzed the sensitivity of frequency and mode shapes to damage to the connection of a steel–concrete composite beam. The sensitivity analysis of the selected modal parameters was performed on the basis of (1) experimental tests on a real-life composite beam, and (2) numerical simulations for the designed numerical model. It should be noted that modeling of multi-material structures, such as steel–concrete composite beams, is a rather complicated process. This primarily relates to difficulties in modeling the connection. However, despite the complexity of the problem, the numerical model of the beam was well matched with the actual beam and provided satisfactory results. It should be added that the verified RFE model can be used in further studies to analyze the behavior of the system.

In the first part of the analyses, concerning the detection of the damage, the sensitivity of the global parameters—the natural frequency and MAC—was determined. Based on the obtained results, it can be concluded that changes in natural frequencies can be useful in identifying damage in the connection plane of beams. The third vibration mode was the most sensitive. The experimental tests for the U2 damage showed an almost 6% change in the frequency. Based on the experimental results and numerical analyses, the conclusion of researchers from Italy (Dilena, Morassi and Rocchetto) that the frequency of flexural vibration is sensitive to damage in the connection plane in steel–concrete composite beams can be confirmed.

MAC analyses showed that it was not effective for identifying damage in the analyzed beams. Increasing the damage level in the beam caused the MAC to either increase or decrease. No close correlation was observed between the increase in the damage degree and the MAC. Thus, it can be concluded that this parameter is not sensitive to damage in the connection plane of beams.

In the second part of the analyses, concerning finding the location of the damage, the sensitivity of the PMAC and the modal curvature that were determined locally for beams was determined. Based on the results obtained for the PMAC, the damage could be effectively located. The PMAC does not indicate the exact place of damage but only the area of its occurrence because it is determined for fragments of the mode shape. Similar to the frequency, it can be observed that the PMAC performed better for higher vibration modes (third and fourth). It should be noted that the method is relatively simple to analyze because it is based only on the analysis of changes in vibration form vectors. It should be noted that when analyzing this characteristic, it is necessary to analyze each mode shape separately. Additionally, each of the analyzed modes should be individually divided into segments for which the PMAC will be determined. The most accurate division seems to be such that the analyzed fragments of modes do not contain the nodal points of these modes.

The results of the performed tests show that localizing damage using the CDF index seems to be effective. The advantage of this method is the fact that the CDF averages the curvature changes for all analyzed vibration modes. Therefore, there is no need to analyze each mode shape separately. Analyzing the results of numerical simulations and actual measurements, it can be concluded that the CDF determined from the numerical analyses indicates the location of damage more accurately, which is most likely related to the increased density of the measurement mesh compared to the experimental tests. It should be noted that the use of measured data for curvature calculations is inherently subject to uncertainty. In practice, for real structures, the curvature of the vibration mode is determined directly from its shape according to relation (2). However, this relation is an approximate formula, which was derived using the finite difference method and is applicable to a sufficiently dense network of measurement points. This was confirmed by the results of the performed analyses. Thus, for effective damage analysis using this method, it is necessary to have measurement equipment with a larger number of sensors.

Further research should be conducted to achieve an accurate and practical method for identifying damage to composite beams. The subject of detecting and pin-pointing the location of damage in composite beams will be continued in future work. Among other things, it is planned to extend the scope of research by analyzing other types of damage and other methods of locating damage.

## Figures and Tables

**Table 1:** Natural frequencies determined in the experimental tests.

Mode Shape		*f_i, exp_*
Flexural vibration	1.	74.68
	2.	172.72
	3.	273.01
	4.	373.40
	5.	472.03
Axial vibration	1.	585.94

**Table 2:** Characteristics used for modeling.

	Parameter	Value
I-beam	*E_s_*	210 × 10^9^ N/m^2^
	*u_s_*	0.3
	*G_s_*	80.77 × 10^9^ N/m^2^
	*r_s_*	7850 kg/m^3^
	*A_s,f_*	6.07 × 10^−4^ m^2^
	*A_s,w_*	7.95 × 10^−4^ m^2^
Concrete slab	*u_c_*	0.2
	*r_c_*	2385.8 kg/m^3^
	*h_c_*	6.06 m

**Table 3:** Identification of stiffness properties of the beam.

*E_c_* (N/m^2^)	*K_X_* (N/m)	*K_Y_* (N/m)
2.89 × 10^10^	2.19 × 10^8^	1.44 × 10^8^

**Table 4:** Comparison of natural frequencies—model and experimental results.

Mode Shape		*f_i, num_* (Hz)	*f_i, exp_* (Hz)	Δ*_i_* (%)
Flexural vibration	1.	74.68	74.93	0.34
	2.	172.72	170.91	1.05
	3.	273.01	275.55	0.93
	4.	373.40	375.06	0.44
	5.	472.03	469.05	0.63
Axial vibration	1.	585.94	585.94	0.00

**Table 5:** Values of the MAC coefficient.

Mode Shape	1 *_flex_*	2 *_flex_*	3 *_flex_*	4 *_flex_*	5 *_flex_*
MAC	0.97	0.92	0.88	0.82	0.72

**Table 6:** Changes in the natural frequency due to damage to the connection—experimental test.

Beam State →	U0	U1		U2	
*i* ↓	*f_i, exp_* (Hz)	*f_i, exp_* (Hz)	Δ*_i_* (%)	*f_i, exp_* (Hz)	Δ*_i_* (%)
1	74.68	73.29	−1.86	73.03	−2.20
2	172.72	167.63	−2.95	165.22	−4.34
3	273.01	259.91	−4.80	257.10	−5.83
4	373.40	357.81	−4.18	354.14	−5.16

**Table 7:** Changes in the natural frequency due to damage to the connection—numerical model.

Beam State →	U0	U1		U2	
*i* ↓	*f_i, num_* (Hz)	*f_i, num_* (Hz)	Δ*_i_* (%)	*f_i, num_* (Hz)	Δ*_i_* (%)
1	74.93	73.96	−1.29	73.92	−1.35
2	170.91	168.89	−1.18	166.41	−2.63
3	275.55	265.71	−3.57	263.00	−4.56
4	375.06	363.58	−3.06	361.40	−3.64

**Table 8:** Changes in the mode shape based on the MAC.

Data →	Numerical Analysis		Experiment	
Beam State →	U1	U2	U1	U2
*i* ↓	$\Delta MAC_{i,num}\;(\%)$		$\Delta MAC_{i,exp}\;(\%)$	
1	0.01	0.01	0.57	0.26
2	0.15	0.14	0.38	0.94
3	1.98	2.02	1.37	1.00
4	2.68	2.66	3.40	5.49

## Data Availability

Not applicable.
